# Comparison of lignin degradation and flavor compound formation in roasted tobacco by two *Bacillus subtilis* strains

**DOI:** 10.3389/fmicb.2025.1538773

**Published:** 2025-01-23

**Authors:** Junmin Wang, Teng Long, Zhenkun Jiang, Wenjun Mu, Mingliang Su, Li Ni, Shunhua Ji, Yuqing Wang, Kangxi Zhou, Renfeng Zhan, Lixuan Nie, Jingjing Li, Xingchuan Hu, Wei He, Wen Zhang

**Affiliations:** ^1^Food Nutrition and Health Research Center, School of Advanced Manufacturing, Fuzhou University, Fuzhou, Fujian, China; ^2^China Tobacco Fujian Industrial Co., Ltd., Xiamen, Fujian, China; ^3^Beijing Life Science Academy, Beijing, China

**Keywords:** roasted tobacco, lignin peroxidase, lignin-degrading bacteria, transcriptome, flavor

## Abstract

Two strains of *Bacillus subtilis* designated YY-10 and BY-2, were isolated from the surface of tobacco and found to be capable of significant lignin degradation. The predominant lignin-degrading enzymes produced by these strains were lignin peroxidase (LiP) and manganese peroxidase (MnP), respectively. A notable distinction was observed in the organoleptic evaluation and volatile flavor compounds, as determined by sensory evaluation and GC–MS analysis. The content of volatile flavor compounds, such as geranylacetone, meglumine trienone B, and meglumine trienone C, was found to be significantly increased in roasted tobacco treated with YY-10. This treatment has been shown to reduce the astringent flavor of the roasted tobacco and improve the aroma, which in turn could improve the quality of the roasted tobacco. Conversely, the quality and aroma levels of the roasted tobacco treated with the crude enzyme solution of the BY-2 strain were reduced. Transcriptome analysis revealed that the expression of genes related to amino acid metabolism, genetic material biosynthesis, and protein synthesis was up-regulated in the YY-10 strain compared with the BY-2 strain, which promoted the biosynthesis of LiP. This study provides a preliminary elucidation of the potential mechanism by which YY-10 enhances the quality of tobacco leaves through lignin-degrading enzyme production, thus establishing a research foundation for the subsequent treatment of waste tobacco raw materials and industrial applications.

## 1 Introduction

Tobacco is a specialized non-edible cash crop. Tobacco leaves usually undergo an aging process before being utilized for industrial applications (Su et al., 2011). In this process, tobacco biomolecules are degraded, aromatic precursors are catalytically transformed, and the sensory quality of tobacco is improved (Liu et al., 2015). Microorganisms actively participate in the aging process, and numerous microorganisms reside on the surface of aged tobacco (Zhang et al., 2020). Under specific conditions, certain microorganisms can degrade proteins, cellulose, lignin, and other substances (Chen et al., 2015; Wang et al., 2017). The flavor of roasted tobacco can be improved by using microorganisms with specific functions to inoculate the surface of tobacco. One of the effective methods to improve flavor without side effects is biofortification by using *Bacillus subtilis*, which can reduce the total carbohydrates and sugars in tobacco and rapidly produce a pleasant aroma after inoculation of its species on the surface of tobacco (English et al., 1967).

Lignin is a complex and diverse polymer found in plant cell walls, and its efficient degradation requires the coordinated action of various microorganisms (Meng and Xue, 2020). In nature, the breakdown of lignin is carried out by a variety of microorganisms, including fungi, bacteria, and actinomycetes. Recent studies have identified several species of *Bacillus bacteria*, such as *Bacillus licheniformis*, *Bacillus subtilis*, *Bacillus thuringiensis*, *Bacillus magaterium*, and *Bacillus cereus*, are capable of degrading lignin or participating in lignin degradation.

The microbial breakdown of lignin into smaller fractions involves the activity of various ligninolytic enzymes (Datta et al., 2017; Falade et al., 2017). These enzymes include laccase (Lac) (Wang et al., 2020), lignin peroxidase (LiP), manganese peroxidase (MnP), cellobiose dehydrogenase (CDH), multifunctional peroxidase (VP), dye decolorizing peroxidase (DyP), aromatic alcohol oxidase (AAO), glycolaldehyde oxidase (GLOX), and hydroquinone reductase (NAHPH) (De Gonzalo et al., 2016; Kersten and Cullen, 2014). These enzymes can decompose aromatic compounds by different mechanisms such as alkyl-aryl cleavage, Cα-Cβ cleavage, aryl ring cleavage, demethoxylation, and cross-linking (Khan and Ahring, 2019). Among these enzymes, laccase, lignin peroxidase, and manganese peroxidase are considered key enzymes in the process of lignin degradation (Furukawa et al., 2014).

Lignin is an important macromolecular substance in tobacco, and its content varies depending on the growth site of the tobacco leaf. Combustion of lignin will make the smoke with heavy green miscellaneous gas, burning smoking will produce burning throat, astringent mouth, dry mouth, and other discomfort. Consequently, the present study screened microorganisms possessing lignin-degrading enzymes from tobacco leaves to reduce their lignin content to a suitable range. This has been shown to have a favorable effect on the enhancement of tobacco flavor as well as quality. In this study, two *Bacillus subtilis* strains, YY-10 and BY-2, were isolated and found to be effective in lignin degradation. Notably, strain YY-10 improved the sensory quality of roasted tobacco through lignin degradation, whereas strain BY-2 adversely affected the quality of roasted tobacco. To elucidate the flavor discrepancies arising from this degradation, a comparative analysis was performed using GC–MS on tobacco samples post-treatment with the two strains. Furthermore, transcriptome sequencing was utilized to decipher potential gene regulatory disparities between YY-10 and BY-2. This research endeavor aims to provide data support and theoretical guidance for the development and utilization of enzymatic formulations that enhance the quality of roasted tobacco, thereby advancing the field of agricultural biotechnology and tobacco processing.

## 2 Materials and methods

### 2.1 Experimental materials

The samples used in this experiment were selected from Yunnan tobacco and provided by the Technical Center of Fujian China Tobacco Industry Co., Ltd.

### 2.2 Main medium

Alkaline lignin medium: Na_2_HPO_4_-12H_2_O 6.2 g, KH_2_PO_4_ 3.0 g, NaCl 0.5 g, NH_4_Cl 1.0 g, alkaline lignin 2.0 g, agar 18 g, deionized water 1 L, adjust pH = 7.0, sterilize at 121°C for 30 min.

Aniline blue medium: tryptone 10 g, NaCl 10 g, yeast extract 5 g, agar 18 g, deionized water 1 L, adjust pH = 7.0, sterilized at 121°C for 30 min. 0.1 g of aniline blue was added when the temperature of the medium was lowered to 60 ~ 70°C after sterilization.

Tryptone soy broth medium (TSB) liquid medium: weigh 30 g of TSB powder, volume to 1 L with deionized water, adjust pH = 7, and sterilize at 121°C for 30 min; for solid medium, add 18 g of agar powder.

Enzyme production fermentation medium: 30 g of tobacco stalks, NaNO_3_ 2.5 g, KH_2_PO_4_ 1 g, MgSO_4_-7H_2_O 0.5 g, NaCl 0.5 g, CaCl_2_ 0.1 g, 1 L of deionized water, adjust pH = 7, 121°C sterilization for 30 min.

### 2.3 Screening of functional strains

Strains were isolated and purified from the surface of tobacco. Guaiacol and aniline blue plates were utilized for targeted screening of strains capable of degrading lignin on the surface of baked tobacco. The strains exhibiting the largest hydrolysis circles were selected for strain preservation and identification (Zhang et al., 2018). Single colonies were then picked and inoculated in TSB medium as seed solution. The seed liquid was inoculated into the enzyme-producing fermentation medium at an inoculum of 10%, and the fermentation broth was incubated at 30°C and 180 rpm for 48 h. The fermentation broth was centrifuged at 12,000 rpm for 10 min, and the supernatant was taken to determine the enzyme activity of the lignin-degrading enzyme.

Lignin peroxidase (LiP): one unit of enzyme activity (U) was defined as the amount of enzyme required to oxidize 1 μmol of veratryl alcohol per minute at pH = 3, 37°C. The reaction system was adjusted according to the method of LiP enzyme activity determination by Tian Linshuang (Tian, 2009), which included 600 μL of resveratrol at a concentration of 10 mmol/L, 1.2 mL of tartaric acid-sodium tartrate buffer (pH = 3) at a concentration of 200 mmol/L, 1.2 mL of crude enzyme solution diluted by a factor of 5, and the reaction was initiated by the addition of 60 μL of hydrogen peroxide at a concentration of 20 mmol/L. The reaction was carried out at 37°C for 2 min. The reaction was started by adding 60 μL of hydrogen peroxide at a concentration of 20 mmol/L, and the reaction was carried out at 37°C for 2 min, and the rate of increase in absorbance at 310 nm was measured in the first 2 min. The crude enzyme solution in a boiling water bath for 15 min was used as a control. Each group of samples was analyzed three times in parallel.

Manganese peroxidase (MnP): one unit of enzyme activity (U) was defined as the amount of enzyme required to oxidize 1 μmol of Mn^2+^ per minute at pH = 4.5 and 37°C. The enzyme activity was determined by using the following method. The reaction system was adjusted according to the method of MnP enzyme activity determination by Zhu et al. (2013), and the system included 3.4 mL of tartaric acid-sodium tartrate buffer (pH = 4.5) at a concentration of 250 mmol/L, 100 μL of MnSO_4_ at a concentration of 1.6 mmol/L, 400 μL of the crude enzyme solution diluted by a factor of 5, and the reaction was initiated by adding 100 μL of hydrogen peroxide at a concentration of 1.6 mmol/L. The reaction was carried out at 37°C. The reaction was initiated by adding 100 μL of hydrogen peroxide at a concentration of 1.6 mmol/L and reacted at 37°C for 2 min. The rate of increase in absorbance at 238 nm in the first 2 min was measured. The crude enzyme solution in a boiling water bath for 15 min was used as a control. Each group of samples was analyzed three times in parallel.

### 2.4 Preparation of the bioaugmentation inoculation

The biofortified inoculation strategy consisted of three steps: (1) growth of the strain, (2) preparation of biofortified, and (3) tobacco fermentation. Firstly, 10 μL of the bacterial solution was taken from a glycerol tube and spread on a TSB solid plate at pH = 7, and incubated at 30°C for 24 h. Single colonies were selected and activated twice on the TSB plate, and the activated single colonies were inoculated into TSB liquid medium at pH = 7, and incubated at 30°C, 180 rpm for 12 h. Secondly, the seed solution was inoculated into an enzyme-producing fermentation medium with an inoculum of 10%, and the fermentation broth was removed after incubation at 30°C, 180 rpm for 48 h. The fermentation broth was then inoculated at 30°C, 180 rpm for 48 h. Then the seed solution was inoculated into 100 mL of enzyme-producing fermentation medium with 10% inoculum, incubated at 30°C and 180 rpm for 48 h. The fermentation broth was extracted and centrifuged at 12000 rpm for 10 min, and the supernatant was taken as the crude enzyme solution. Finally, 2.5 mL of the crude enzyme solution was sprayed evenly on the surface of 50 g of roasted tobacco with a portable spray gun, during which it was ensured that there was no aroma or odor on the hands so as not to affect the results of sensory evaluation, and the fermentation was carried out at 37°C for 4 h. After the completion of the fermentation, the tobacco was taken out and laid flat on a pallet to be heated uniformly and then baked at 135°C for 70 s for the inactivation of enzymes, and then placed in the constant-temperature and constant-humidity oven to equilibrate the water for 48 h. The water and the best sensory evaluation of the fermentation broth were performed in the same volume of water. The same volume of water and the crude enzyme solution after the inactivation of the sample group with the best sensory evaluation (boiling water bath for 15 min) were used as controls.

### 2.5 Sensory evaluation test

After uniform distribution of the roasted tobacco, the tobacco was put into the empty sleeve with a cigarette lighter and made into sticks with a mass of 1 g (± 0.1 g), which were randomly numbered and then scored by 8 experts from the Technical Center of Fujian China Tobacco Industry Corporation for sensory evaluation. The experts were required to score the aroma, aroma volume, fineness, off-gassing, irritation, sweetness, flavor, aftertaste, strength, and concentration of the roasted tobacco after different treatments, and take the average value.

### 2.6 Determination of the relative content of lignin and flavor components in roasted tobacco

The acid-soluble lignin and acid-insoluble lignin contents were determined concerning the assays described by Chu and Li (2016) and Chu and Li (2015) 0.5 g of roasted tobacco was weighed and loaded into the injection bottle, and then 3 μL of 2 mg/mL phenethyl acetate solution was added as the internal standard, and analyzed by gas chromatography–mass spectrometry (GC–MS), and the resulting spectra were searched and characterized by computerized spectral libraries (NIST14), and those with the matching scores higher than 60% were selected, and the relative contents of the volatile flavors in the roasted tobacco were calculated with the method of the internal standard. The content of volatile flavor substances (μg/g) = (c × v × A1) / (A0 × m0) In the formula: c is the concentration of the internal standard, mg/mL; v is the volume of the internal standard, μL; A1 is the peak area of volatile substances; A0 is the peak area of the internal standard; and m0 is the mass of tobacco, g. The relative content of volatile flavor substances in roasted tobacco was calculated by the internal standard method.

GC–MS conditions: HP-5 ms column (60 m × 0.25 mm × 0.25 μm), helium as carrier gas, flow rate 1 mL/min; no split injection; inlet temperature: 250°C; heating program: starting temperature 50°C, hold for 2 min, rise to 180°C at 2°C/min, then rise to 280°C at 5°C/min, hold for 10 min; post-run temperature 280°C, post-run time 5 min; solvent delay 5.5 min. Quadrupole temperature 150°C; EI ionization source, electron energy 70 eV, ion source temperature 230°C, mass scan range m/z 33 The temperature of the four-stage rod was 150°C; EI ionization source, electron energy 70 eV, ion source temperature 230°C, mass scanning range m/z 33–400.

### 2.7 RNA extraction, sequencing, and transcriptome analysis

The RNA samples of *B. subtilis* YY-10 and BY-2 were entrusted to Shanghai Meiji Biomedical Technology Co. The total RNA was extracted from the tissue samples, and the concentration and purity of the extracted RNA were examined by Nanodrop 2000, the RNA integrity was examined by 1% agarose gel electrophoresis, and the RIN value of RNA was determined by Agilent 2,100. The library was sequenced using the NovaSeq 6,000 sequencing platform. Bioinformatics analysis was performed based on the generated data.

### 2.8 RT-PCR analysis

RNA reverse transcription synthesizes cDNA.

The qPCR reactions were performed according to the qPCR reaction kit (NovoStart® SYBR qPCR SuperMix Plus). The cDNA samples were diluted and used as templates on the machine using rpoB as an internal reference gene. The primer sequences for the key genes were designed from Appendix 3 and synthesized by Sangon Biotech. qRCR reaction procedure: pre-denaturation 95°C (60 s); denaturation 95°C (20 s), annealing 55°C (20 s), extension 72°C (30 s), a total of 40 cycles were performed.

### 2.9 Statistical analysis

Data were processed using Excel (results expressed as mean ± standard deviation) and visualized on radar charts and bar charts with GraphPad Prism 8.0. The data processing results were presented as mean ± standard deviation. Significance was assessed using Duncan’s test with SPSS 22.0 software, and the data were visualized using GraphPad Prism 8.0 software. Data from transcriptome sequencing were processed and visualized using the BioSignal Cloud Platform by Meiji Bio.

## 3 Results

### 3.1 Screening of lignin-degrading bacteria on the surface of roasted tobacco

**Figure 1:**
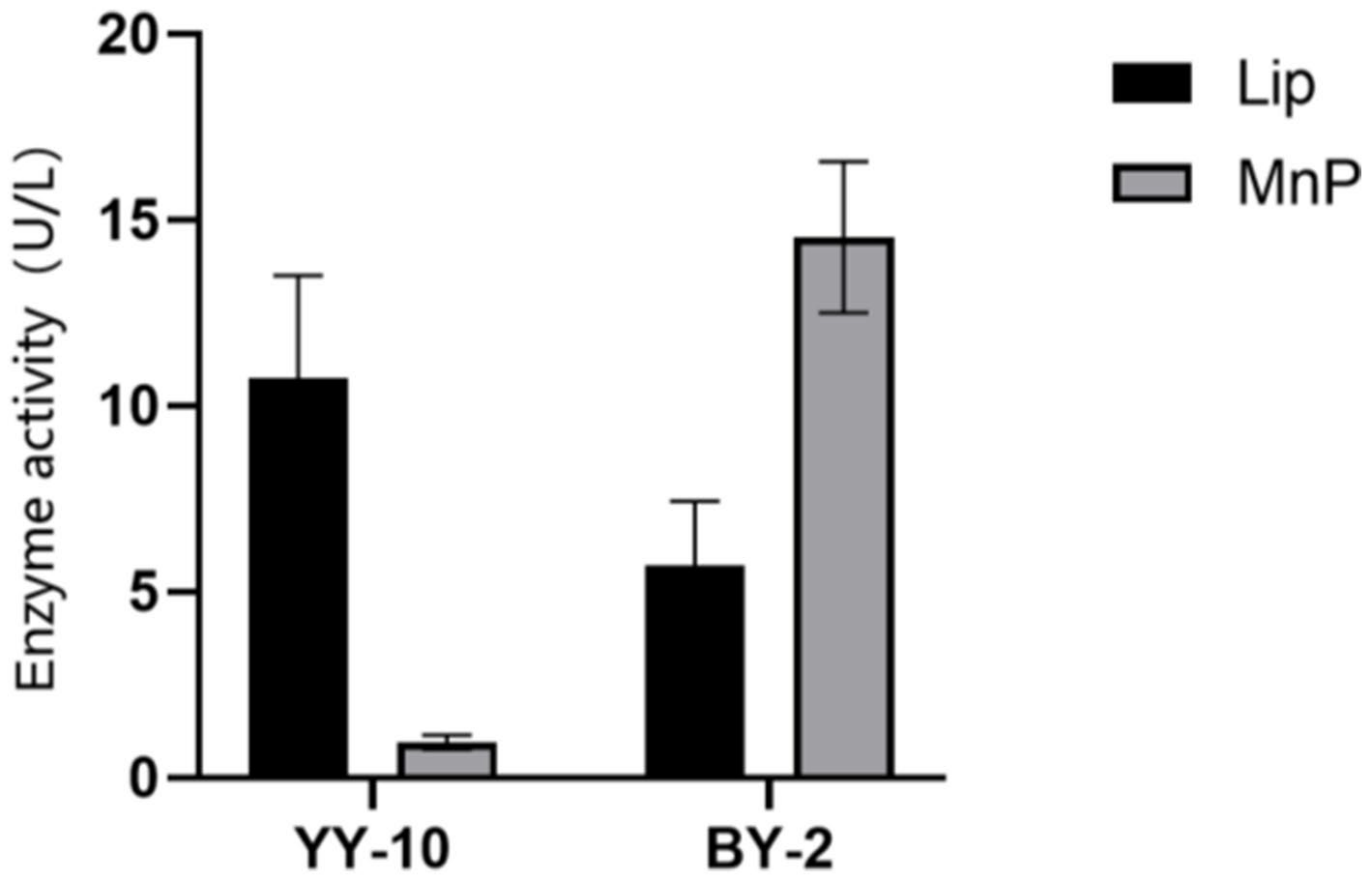
Lignin peroxidase (LiP) and manganese peroxidase (MnP) activities of *Bacillus subtilis* YY-10 and BY-2 strains.

Two lignin-degrading strains were obtained from purified bacteria on aniline blue plates, screened by their hydrolysis circle-to-colony diameter ratio on the plates (Supplementary Table S1), originally isolated from roasted Yunyun 87 and CB-1 tobacco on alkaline lignin plates. The 16S rDNA sequences were analyzed on NCBI via BLAST, confirming them as *B.subtilis* YY-10 (GenBank: SUB12195781) and *B.subtilis* BY-2 (GenBank: SUB12195784). The strains were inoculated into enzyme-producing fermentation media, and enzyme activities were assayed over 48 h. Notably, Lac activity was undetected, while LiPase and MnPase activities peaked at 11.16 U/L for YY-10 and 14.53 U/L for BY-2, respectively (Figure 1).

### 3.2 Sensory evaluation results and chemical composition analysis

**Figure 2:**
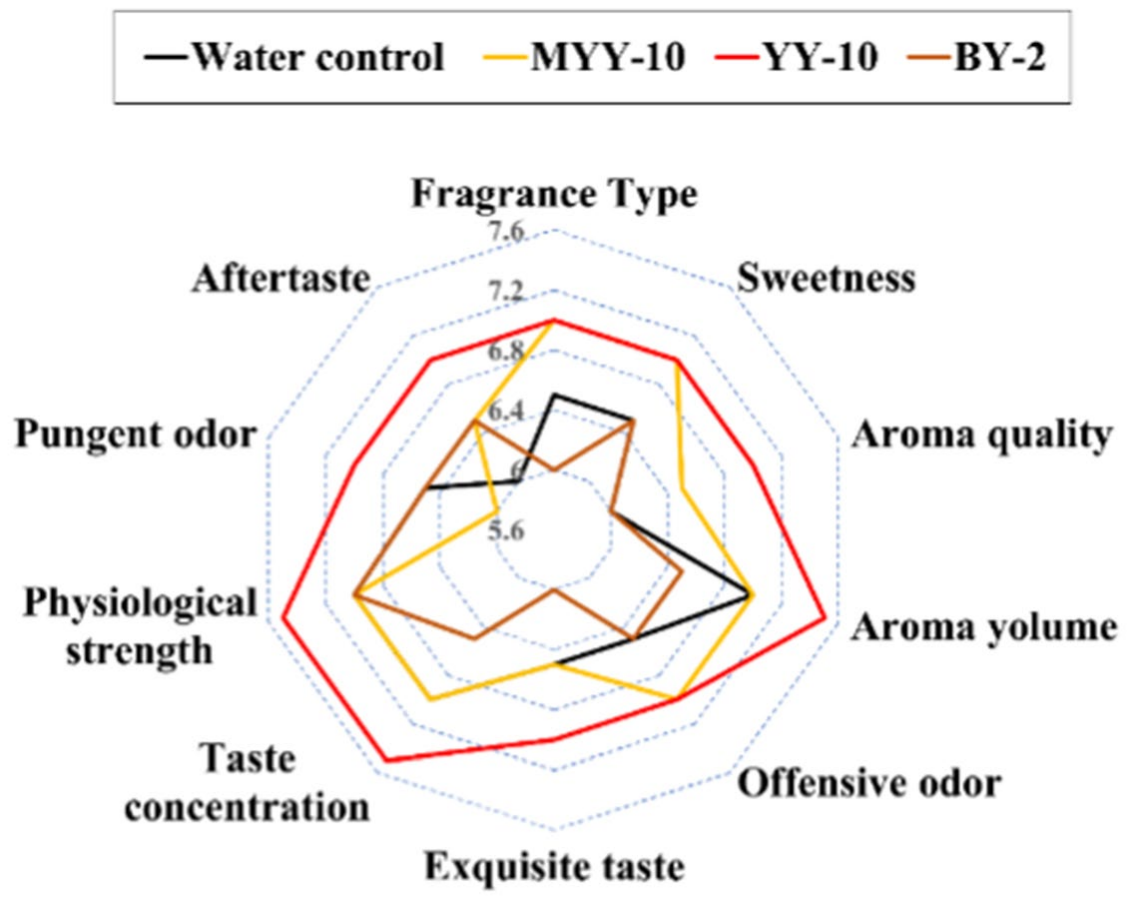
Sensory evaluation results of roasted tobacco in different treatment groups. MYY-10: YY-10 fermentation crude enzyme solution inactivation treatment group. YY-10: YY-10 fermentation crude enzyme solution treatment group. BY-2: BY-2 fermentation crude enzyme solution treatment group. The same is below.

Application of the 24-h fermented crude enzyme solutions from YY-10 and BY-2 to roasted tobacco revealed distinct sensory effects: YY-10 > water control > BY-2 (Figure 2). YY-10 significantly enhanced sweetness, aroma, and fragrance while mitigating undesirable odors and irritation. In contrast, BY-2 showed no improvement. The inactive enzyme solution from YY-10 had no impact, suggesting strain metabolites and medium composition were not flavor determinants. Despite both being *Bacillus subtilis*, LiP-producing YY-10, and MnP-producing BY-2 exhibited markedly different effects on tobacco flavor (Figure 2).

**Figure 3:**
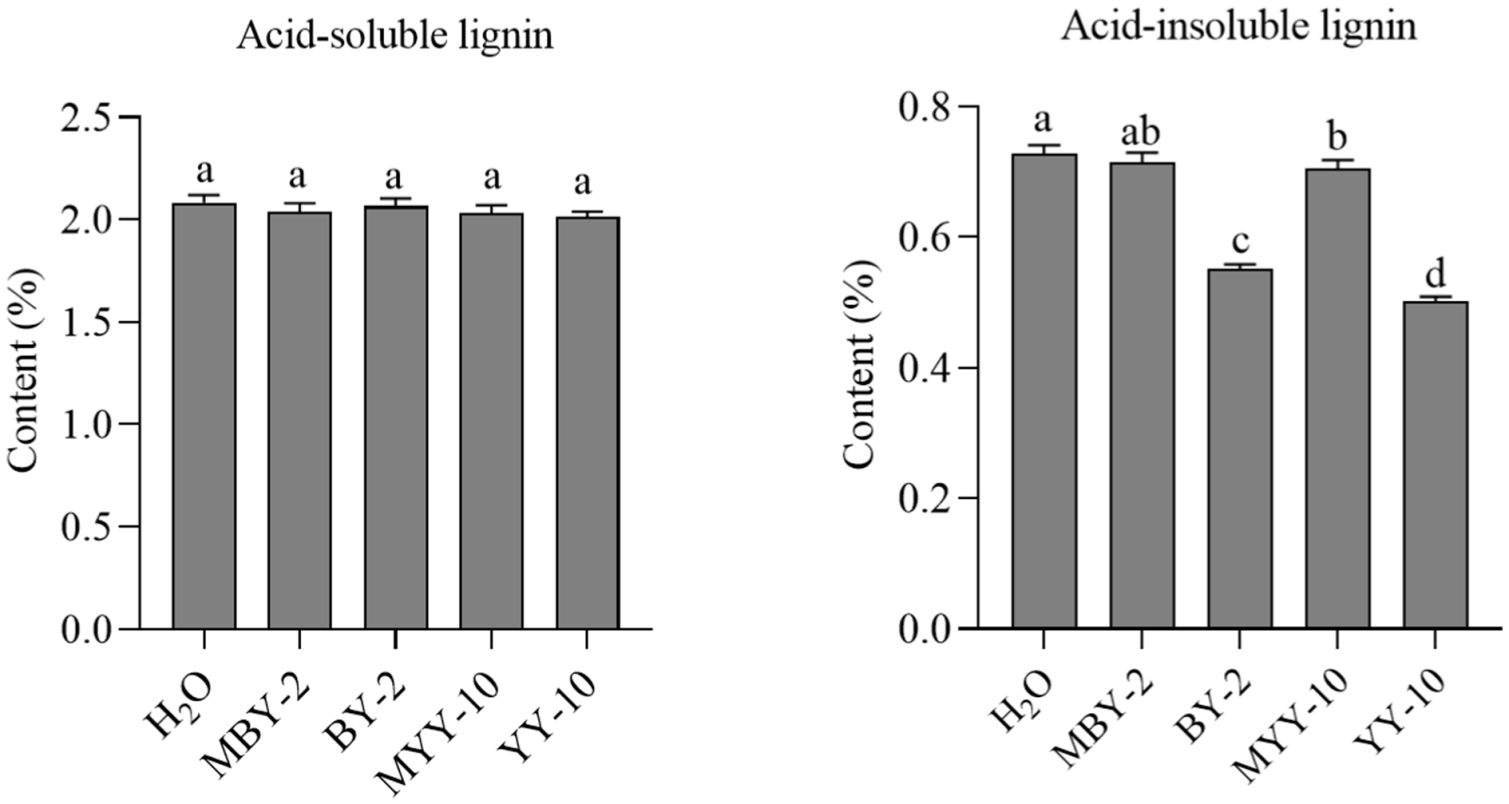
Changes of lignin content in roasted tobacco after different treatments.H_2_O: water treatment group. MBY-2: BY-2 fermentation crude enzyme solution inactivation treatment group. The same is below.

The fermented crude enzyme solution of both strains, BY-2 and YY-10, failed to significantly reduce the content of acid-soluble lignin in roasted tobacco. However, both solutions were able to degrade acid-insoluble lignin in the tobacco, with the fermented crude enzyme solution of strain YY-10 exhibiting a superior degradation effect compared to that of BY-2 (Figure 3).

**Figure 4:**
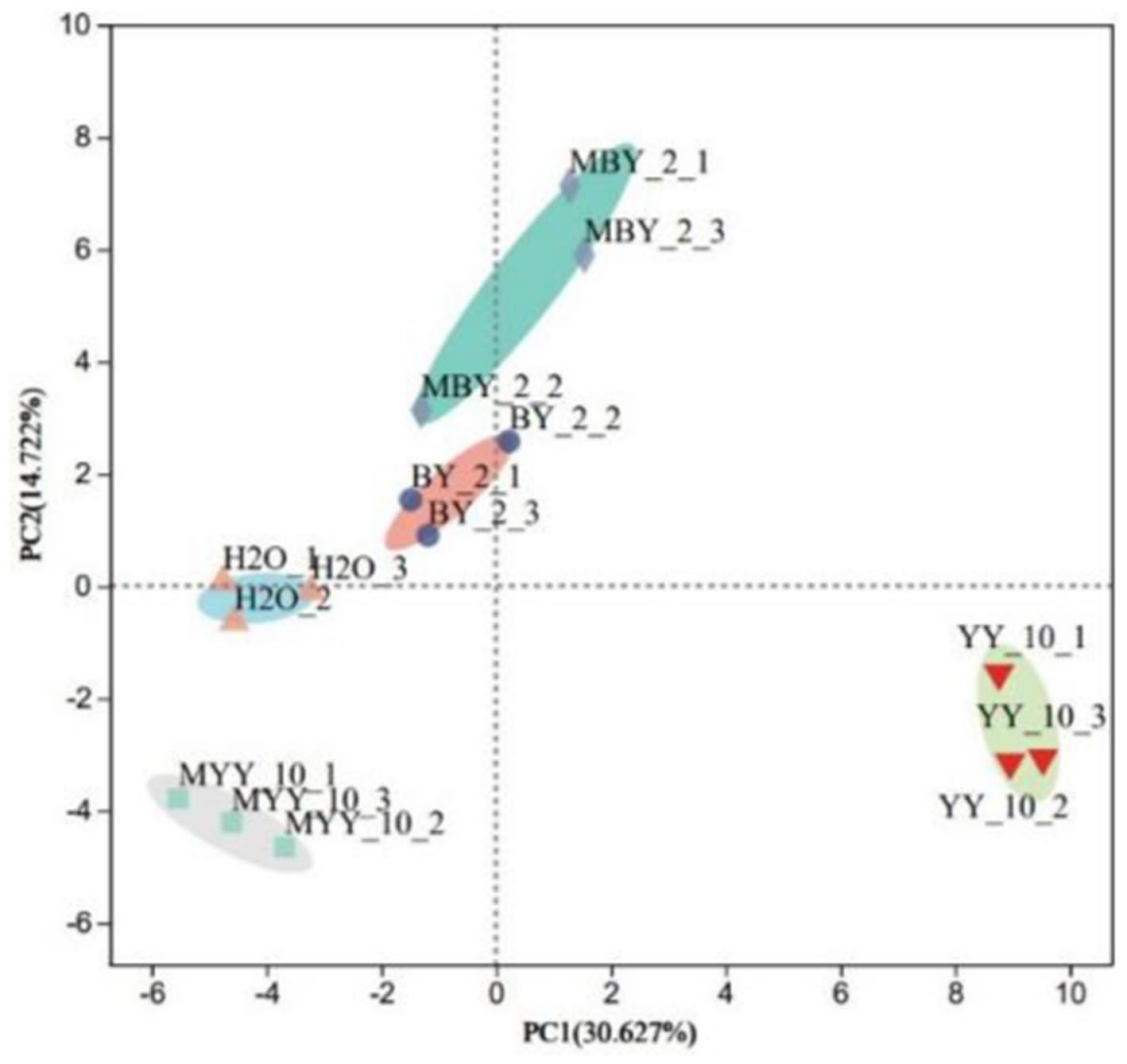
Principal component analysis of volatile substances in roasted tobacco after different treatments.

The content of volatile flavor substances in roasted tobacco was analyzed by GC–MS. The Principal Component Analysis (PCA) comprehensively evaluated the types and contents of these substances in tobacco treated with different methods. The PCA scatter plots (Figure 4) revealed substantial differences in volatile compounds between the BY-2 and YY-10 fermented crude enzyme solution-treated groups, as well as the control groups, indicating significant variations in the volatile substances of the roasted tobacco.

**Figure 5:**
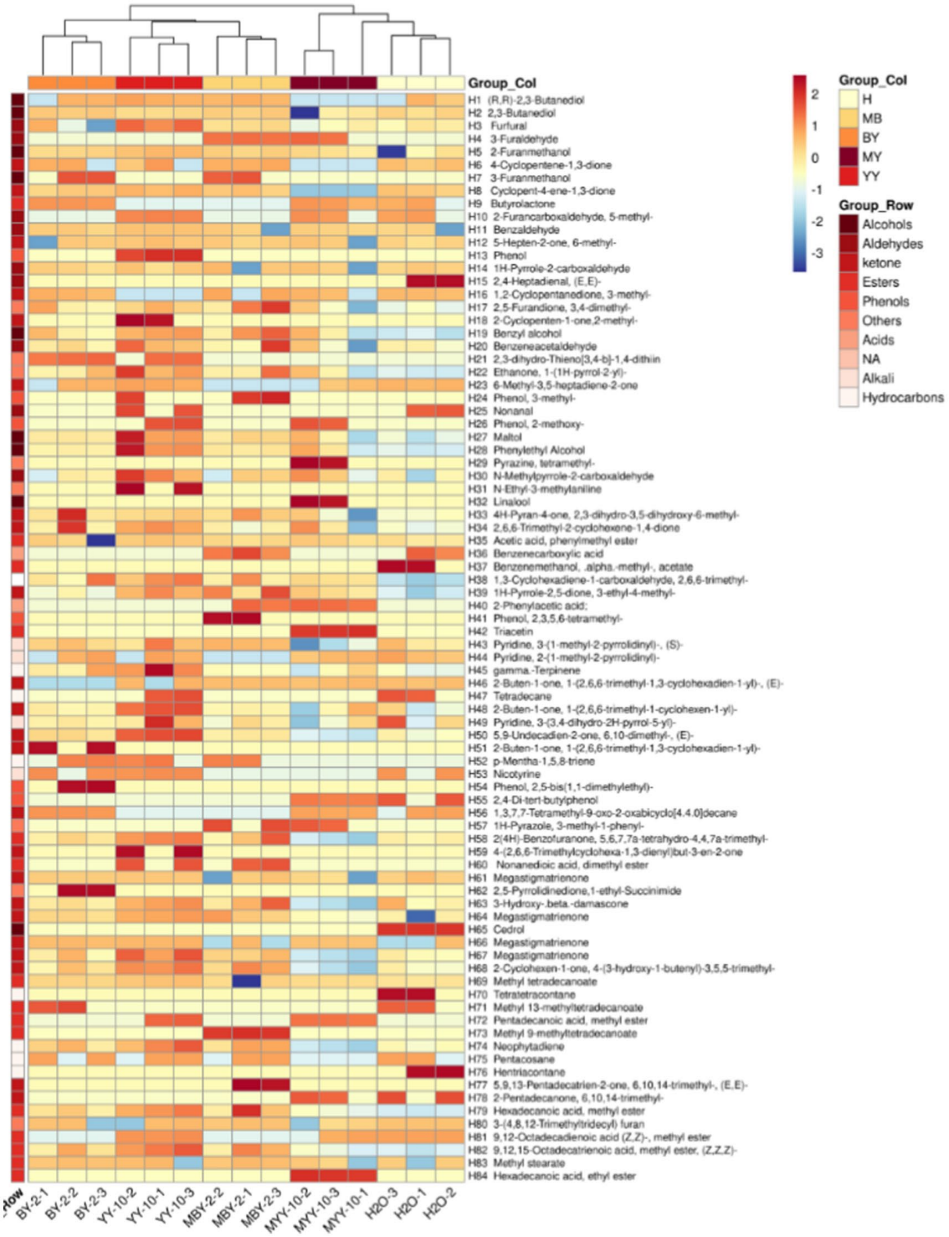
Clustering heat map of volatile substances in roasted tobacco after different treatments.

**Figure 6:**
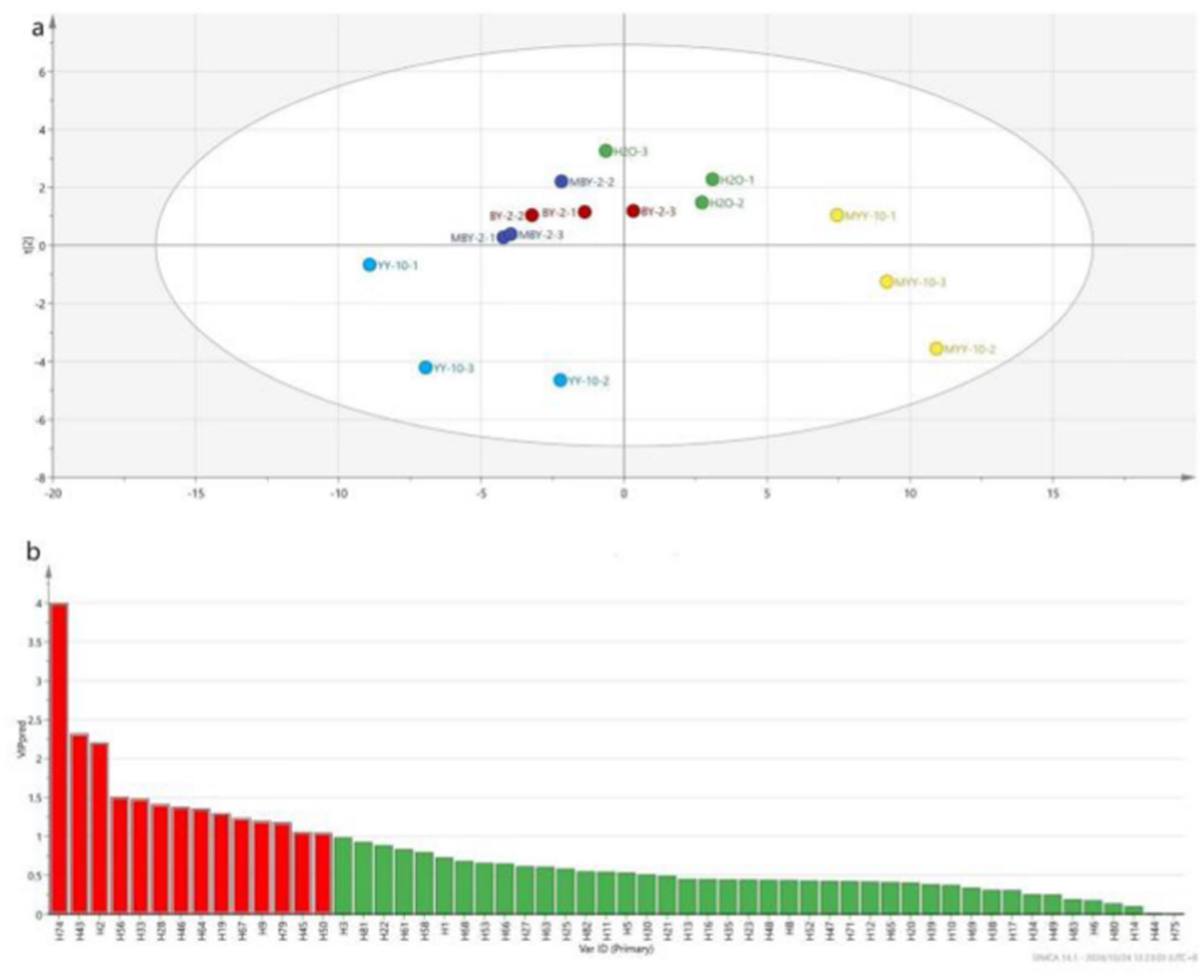
PLS-DA model scores of volatile flavor substances of roasted tobacco after different treatments **(A)** and VIP value of volatile flavor compounds **(B)**.

As shown in Figure 5, a total of 84 volatile flavor substances were identified by GC–MS, comprising 9 alcohols, 23 ketones, 10 aldehydes, 6 phenols, 15 esters, 2 acids, 4 bases, 7 hydrocarbons, and 8 other substances. Ketones are the primary components that form the subtle aroma of tobacco leaves. Alcohols provide its characteristic sweet, fruity, and herbal aroma. Esters can affect the aroma and flavor of the tobacco leaves and add the pure and soft feeling of the smoke (Li et al., 2023; Wu et al., 2021; Xu et al., 2020; Liu et al., 2023; Liu, 2014). The VIP values of each volatile flavor substance in roasted tobacco from different treatment groups were calculated by SIMCA software (Lan et al., 2022). The volatile aroma substance content of VIP > 1 was analyzed in Figure 6. To further investigate the differences in volatile aroma substance content between treatment groups. Effects of different volatile flavor substances on the aroma characteristics of roasted tobacco are shown in Supplementary Table S2.

The volatile flavor substance content of the YY-10 fermented crude enzyme solution treatment group was significantly different from that of the water control and the BY-2 fermented crude enzyme solution treatment group among the substances with a *p* value of >1.

Different volatile flavor substances confer different aroma characteristics to the roasted tobacco. Benzene ethanol has a sweet, rose-like flower scent (Liu et al., 2022), and Geranyl acetone gives the smoke a floral and woody aroma (Lv et al., 2012; Liu et al., 2017). Megastigmatrienone B and Megastigmatrienone C confer a hay-like sweet aroma to the roasted tobacco, which can increase the smoke aroma and reduce irritation of the roasted tobacco (Zhang et al., 2018).

**Figure 7:**
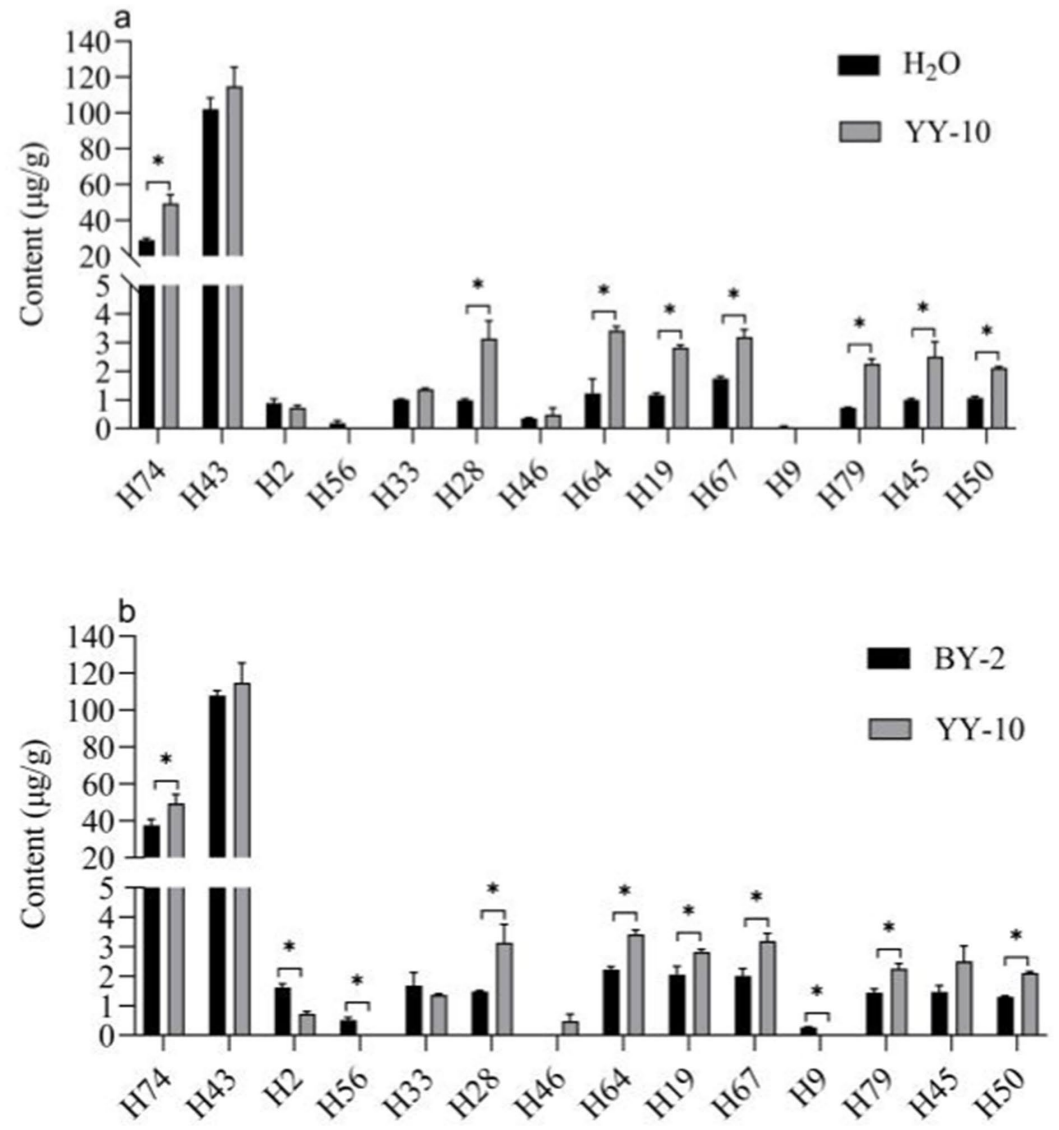
Significance analysis of volatile flavor content in different treatment groups **(A,B)**. “*” indicates significant differences at the 0.05 level (*p* < 0.05); BY-2: BY-2 enzyme-treated group; YY-10: YY-10 enzyme-treated group.

It could be the reason why the fermented crude enzyme solution of the YY-10 strain treated with roasted tobacco increased the sweetness of roasted tobacco, had a better aroma, reduced the miscellaneous air and irritation, and significantly improved the flavor of roasted tobacco (Figure 7).

### 3.3 Transcriptome analysis

The gene regulatory mechanisms of two *Bacillus subtilis* strains, YY-10 and BY-2, involved in lignin degradation were investigated using transcriptome sequencing. Meanwhile, the expression of differential genes of different strains was explored at the transcriptome level, and the enrichment pathways of differential genes were analyzed. The study revealed the potential mechanism of high LiPid production of strain YY-10, which provides more options for the industrial use of the strain for enzyme production to improve the flavor of roasted tobacco. The judgment threshold for the average error rate of sequencing bases was 0.1%, and the error rates of all six groups of samples were below 0.026% (Supplementary Table S3). The results indicated that the sequencing results were satisfactory, the samples were not contaminated, and the sequencing results could be used for subsequent assembly and transcriptome analysis.

**Figure 8:**
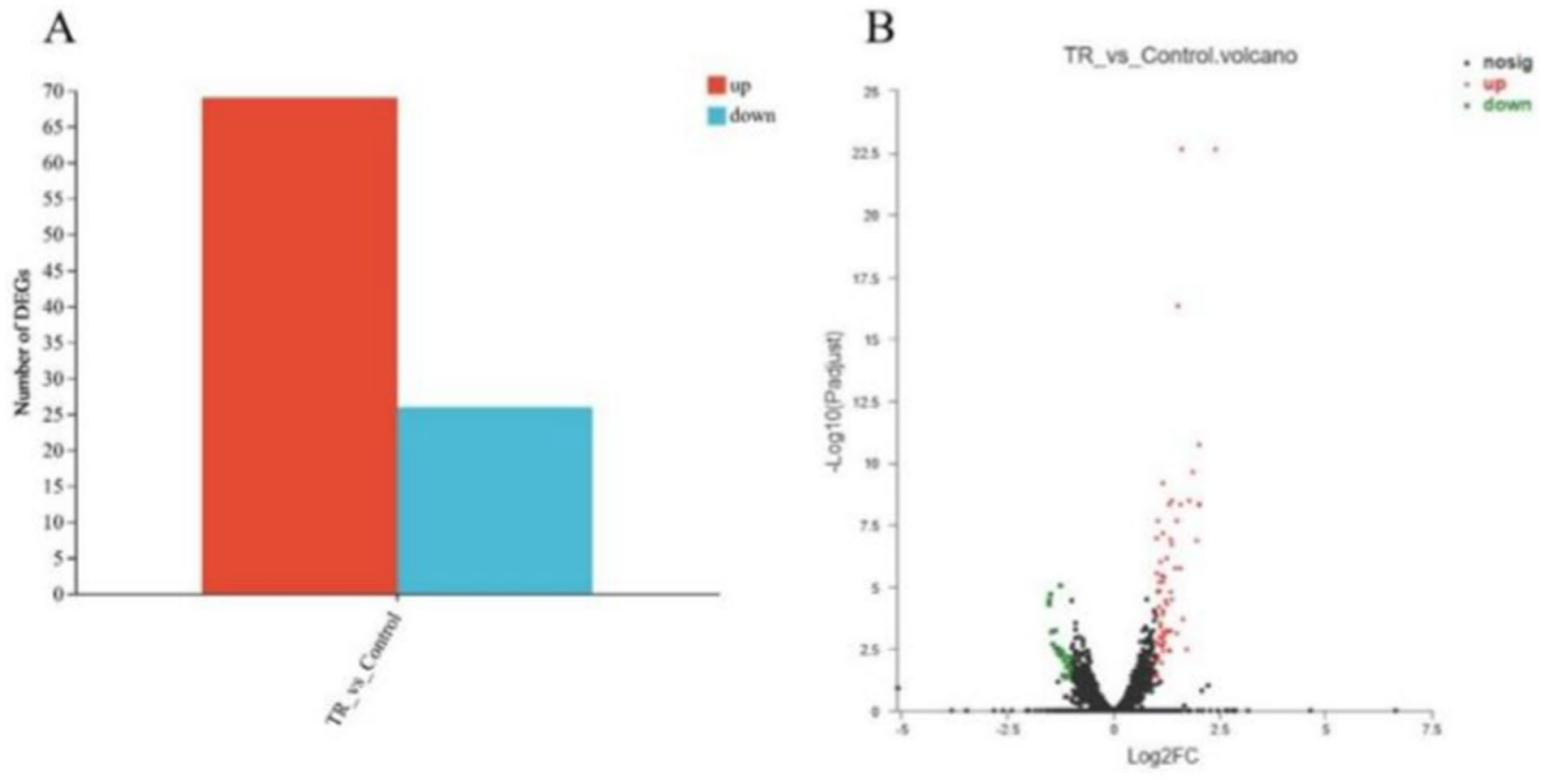
Histogram **(A)** and a volcano plot **(B)** of differentially expressed genes.

The genes with significant differences among the samples were selected by setting the two screening conditions of *p* < 0.05 and multiplicity of up/down-regulated differences >2. A total of 95 genes were differentially expressed as shown in Figure 8A, of which 69 were up-regulated genes and 26 were down-regulated genes. The significant difference between the two sets of sample data can be visualized by plotting the volcano plot (Figure 8B).

### 3.4 Differential gene GO enrichment analysis

**Figure 9:**
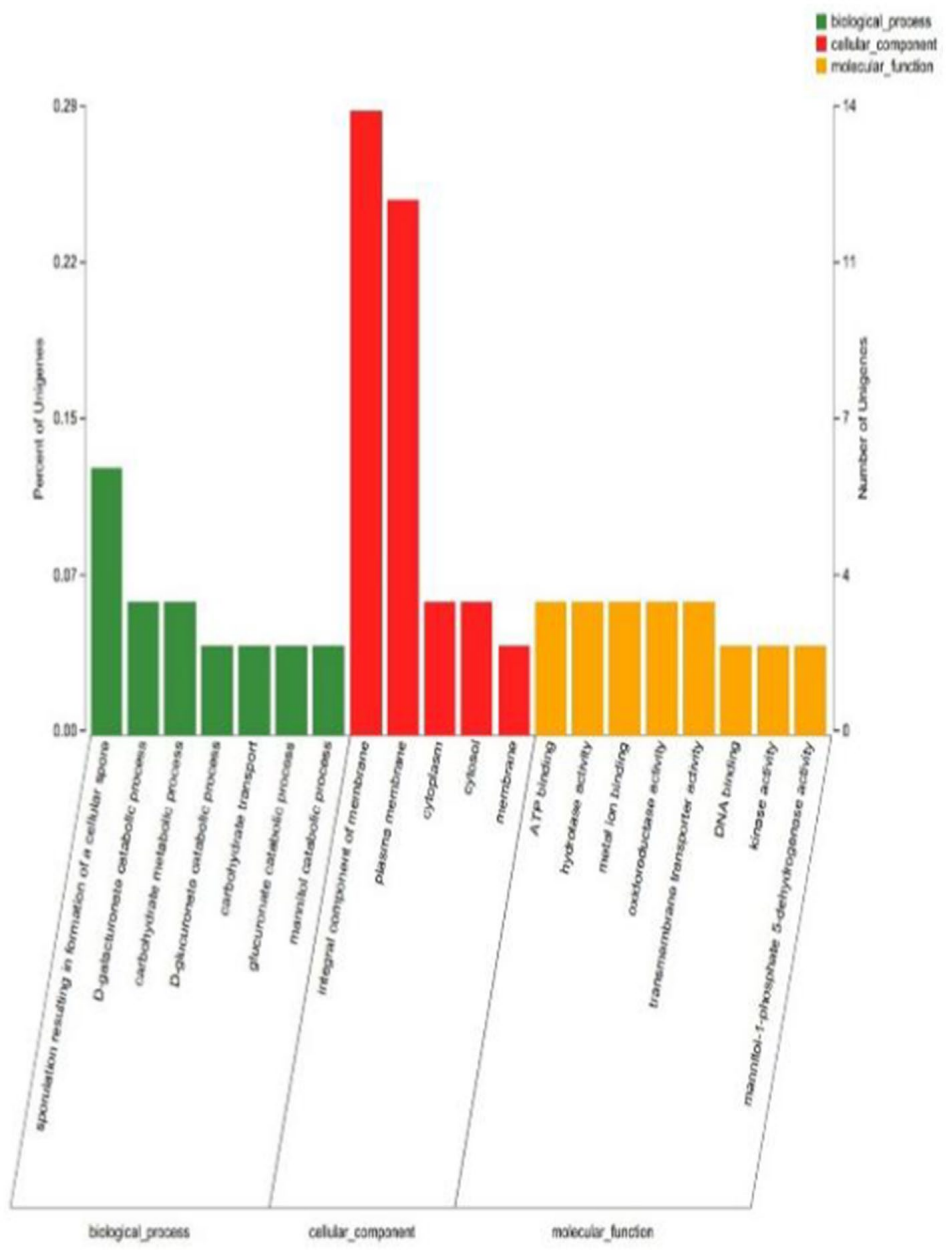
GO annotation classification diagram of differentially expressed genes.

GO functional annotation was performed for YY-10 and BY-2 strains, with a total of 95 differentially expressed genes compared, of which 75 could be annotated to the GO database. The GO classification of the differentially expressed genes (Figure 9) showed that 75 differentially expressed genes were annotated into three major categories with 20 secondary entries. Among them, 20 were annotated as biological processes, 34 as cellular components, and 21 as molecular functions.

**Figure 10:**
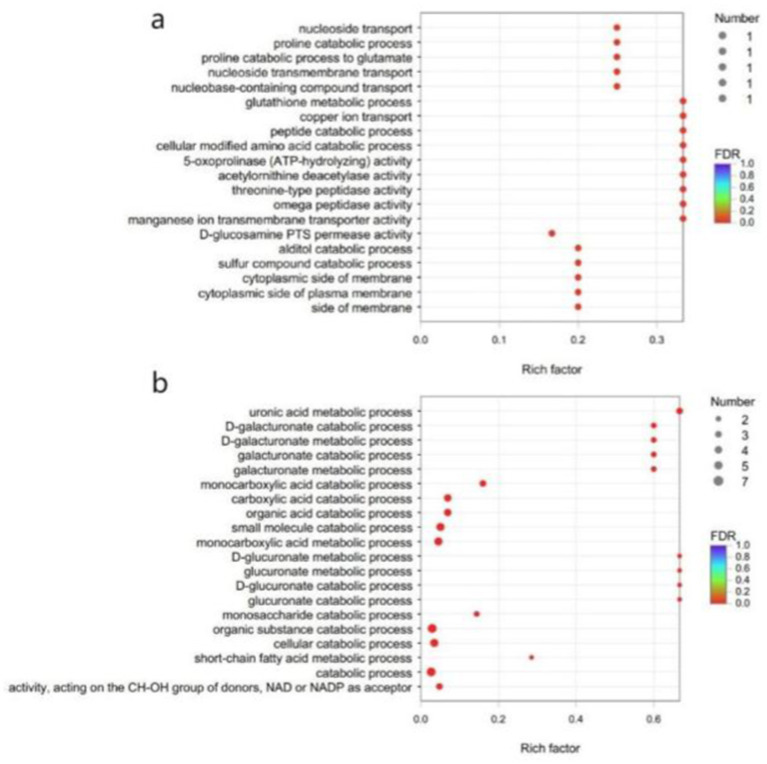
GO enrichment analysis. **(A)** up-regulated genes **(B)** down-regulated genes. The vertical axis represents the GO term, and the horizontal axis represents the enrichment ratio, i.e., the ratio of the number of genes in the gene set annotated to the GO term to the number of genes in all genes annotated to the GO term. The larger the enrichment ratio, the greater the degree of enrichment. The size of the dots indicates the number of genes in the GO term, and the color indicates the significant degree of enrichment. The redder the color, the more significantly enriched the GO term is.

Due to the small number of annotatable differential genes in the GO database, GO enrichment analysis of the differential genes of *B. subtilis* YY-10 and BY-2 was performed using a *p* value corrected to <0.05 as the critical value. Eight genes were up-regulated and seven genes were down-regulated in *B. subtilis* YY-10 (Figure 10).

Fifteen key genes from the GO enrichment analysis were selected for qRT-PCR to assess the validity of the RNA-Seq data. The relative expression of key genes in *B. subtilis* YY-10 and BY-2 was determined by RT-qPCR. The expression trends of the genes except *uxaA* were generally consistent with the transcriptome results, which confirmed the reliability of the RNA-seq expression data. Among the genes with extremely significant high expression *hpxW*, *pxpA* is associated with the *γ*-glutamyl cycle, which contributes to the uptake of amino acids by cells from the culture environment. The significantly highly expressed genes include the genes *copO* and *pxpG*, which are related to the metal ion transmembrane transport pathway, and the *mtlF* and *mtlD*, which are related to the synthesis of intracellular fructose. The significant up-regulation of these genes in *B. subtilis* YY-10 may be closely related to the high LiP production of the strain.

## 4 Discussion

A total of 40 bacterial strains were screened from the surface of roasted tobacco, among which two strains had strong enzyme production ability. The enzyme activity of the lignin-degrading enzyme and the results of the sensory evaluation showed that *Bacillus subtilis* YY-10 mainly produced lignin peroxidase (LiP), and its fermented crude enzyme solution could improve the quality of roasted tobacco, which could make the roasted tobacco more full-flavored, better aroma, purer smoke, and more natural smoke flavor. *B. subtilis* YY-10 was compared with *B. subtilis* BY-2, a strain that failed to improve the flavor of roasted tobacco, to investigate the effects of the fermentation broth of the two strains on the contents of lignin and volatile flavor substances in roasted tobacco. The results showed that the degradation of acid-insoluble lignin by YY-10 fermentation broth was better than that by BY-2 fermentation broth, and the reduction of lignin content in roasted tobacco was conducive to the alleviation of off-flavor and irritation of roasted tobacco.

The results of GC–MS showed that compared with the water-treated group, the BY-2 fermented crude enzyme solution-treated group, and the YY-10 fermented crude enzyme solution inactivated enzyme treatment group, the effect of YY-10 fermented crude enzyme solution on the surface of roasted tobacco could degrade the lignin content in roasted tobacco by various methods, resulting in a significant increase in volatile flavor substances with VIP values >1 such as Geranyl acetone, Megastigmatrienone B and Megastigmatrienone C. The increase in the content of these substances can improve the amount aroma of roasted tobacco, so that the roasted tobacco has a variety of aromas such as floral, fruity, grassy, etc., and further harmonize the aroma of the roasted tobacco, making the smoke more mellow and improve the quality of the roasted tobacco.

**Figure 11:**
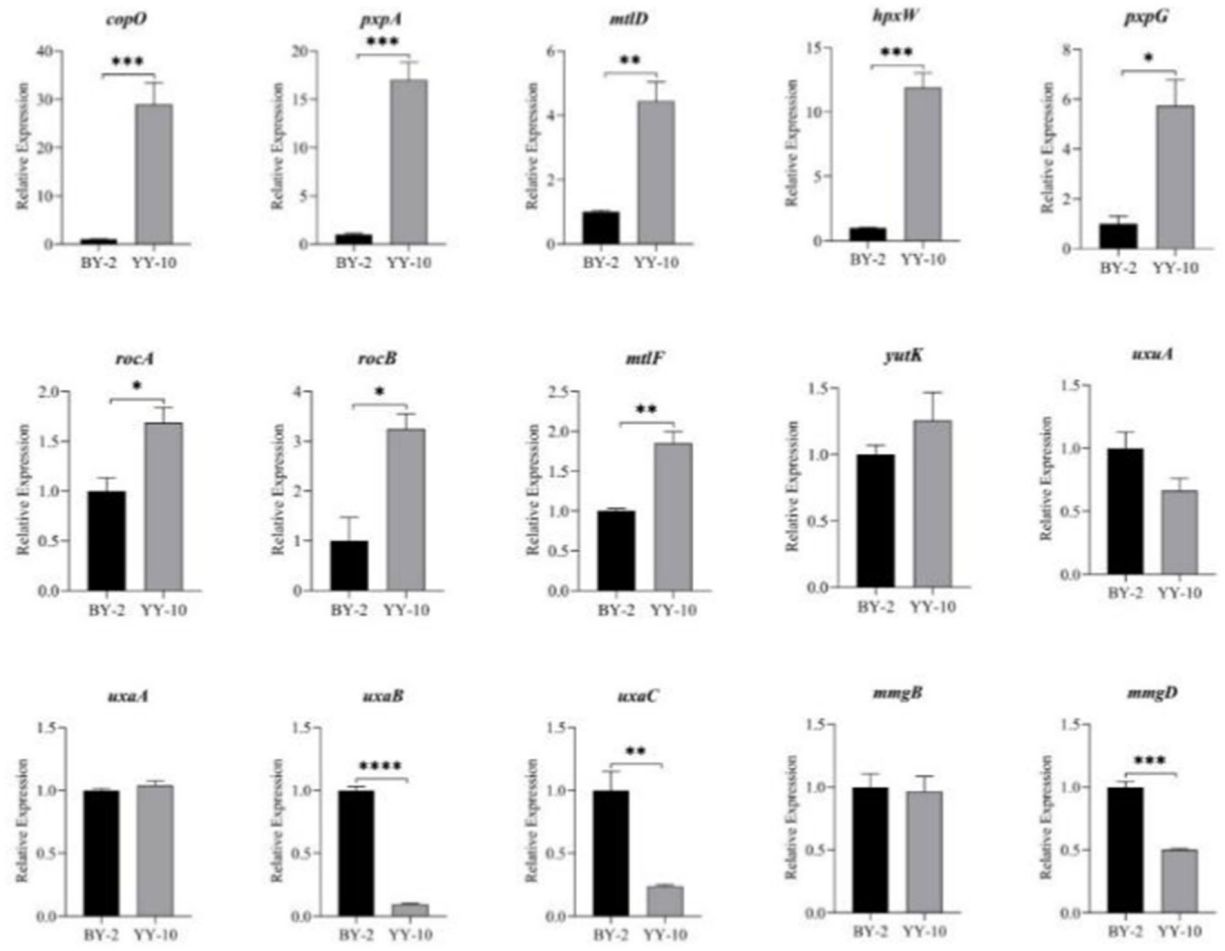
Expression of key genes during the fermentation process. “*” indicates a significant difference at the 0.05 level (*p* < 0.05), “**” indicates a significant difference at the 0.01 level (*p* < 0.01), “*** “indicates a significant difference at the 0.001 level (*p* < 0.001), and “****” indicates a significant difference at the 0.0001 level (*p* < 0.0001).

**Figure 12:**
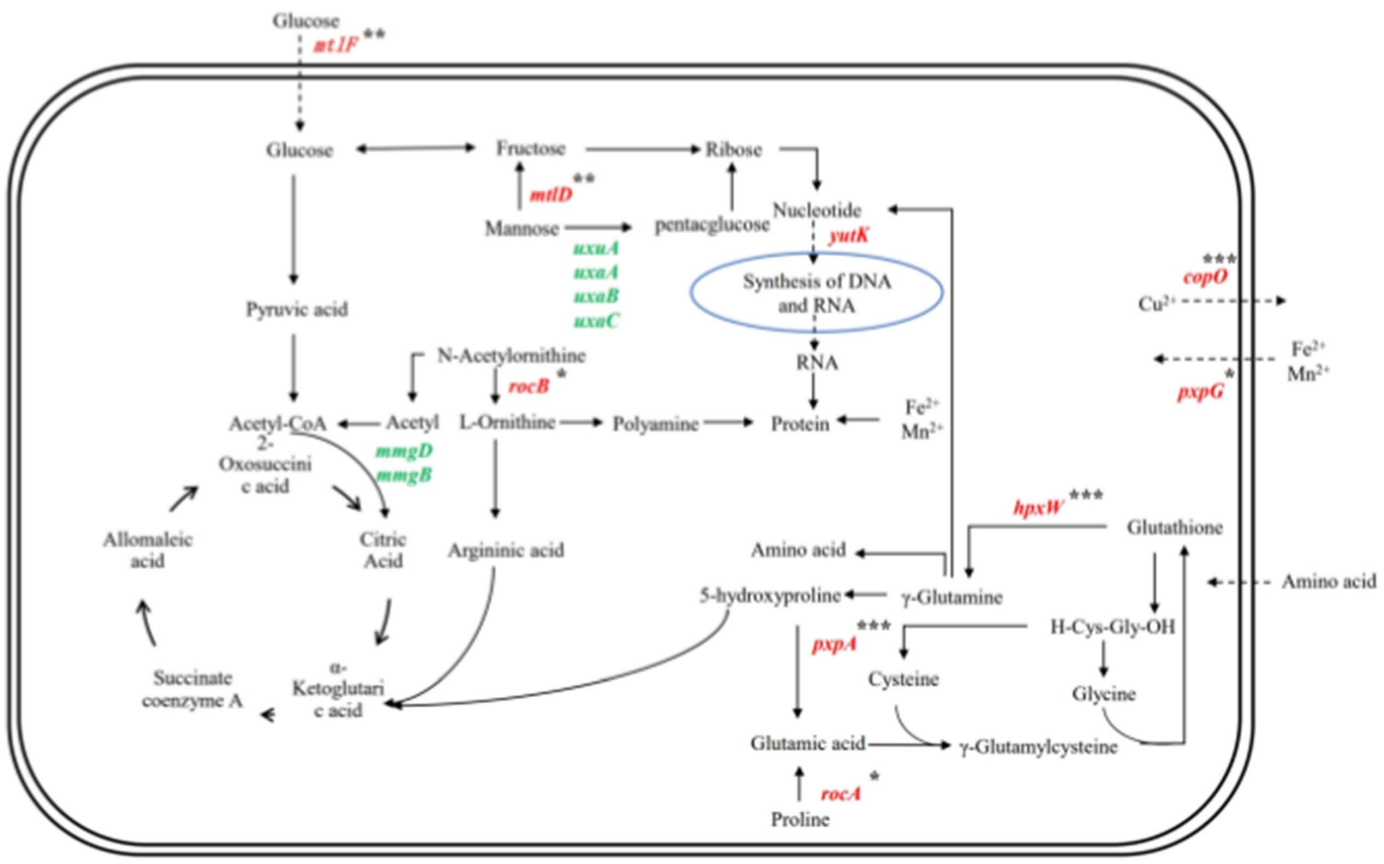
Gene network regulation map related to the secretion of LiP by YY-10 strains. Red color indicates up-regulated genes and green color indicates down-regulated genes.

As shown in Figure 11, *Bacillus subtilis* YY-10 may enhance LiP production by accelerating the uptake of glucose and amino acids from the culture environment and accelerating the synthesis of genetic material and proteins, as shown in Figure 12.

The expression of *hpxW*, a gene related to the glutathione metabolic process, and *pxpA* (Niehaus et al., 2017), a gene related to 5-oxoprolinase (ATP-hydrolyzing) activity, were up-regulated, which promoted the *γ*-glutamyl cycle-catalyzed glutathione production. Catalyze the intracellular release of γ-glutamyl groups of glutathione bound to extramembrane amino acids and brought into the cell, contributing to the uptake of amino acids by the cell from the culture environment (Meister, 1982).

The gene *mtlF* (Reiche, 1998), associated with the D-glucosamine PTS permease activity in the YY-10 strain, along with the gene *mtlD*, which encodes erythritol-1-phosphate 5-dehydrogenase involved in alditol catabolism, are both upregulated. This upregulation enhances the functionality of the PTS system and facilitates the conversion of mannose to fructose. Subsequently, fructose can be further transformed into ribose, thereby promoting nucleotide synthesis and increasing RNA levels necessary for protein synthesis (Liu et al., 2020; Liu and Li, 2010).

The combination of the above pathways suggests that the YY-10 strain has an increased ability to accumulate RNA and amino acids in the cytosol compared to the BY-2 strain, which is beneficial to protein synthesis. The expression of *copO* and *pxpG* genes in the transmembrane transport pathway of metal ions was up-regulated, which facilitated the transfer of Cu^2+^ and Fe^2+^. LiP is a ferroheme-containing peroxidase, which produces metal ions for LiP synthesis through the biochemical reaction of cycling of Fe^2+^ and Fe^3+^ in the cell (Liu et al., 2023), and the increase of Fe^3+^ could increase the activity of lignin peroxidase (Merino et al., 2021). Cu^2+^ inhibits the production of LiP by the strain and also has the activity of LiP. Therefore, the YY-10 strain is more capable of producing lignin peroxidase than the BY-2 strain.

The LiP is considered to be the most efficient enzyme for lignin degradation, having a very low optimum pH compared to *MnP*, close to pH 3.0, showing non-specificity for substrates in general, and can oxidize phenolic units, non-phenolic lignin units and a range of compounds with redox potentials higher than 1.4 V in the presence of hydrogen peroxide as an electron acceptor to produce some flavor-related volatiles (Kirk and Farrell, 1987).

In contrast, the Mn^3 +^ produced by MnP must be stabilized by chelation with organic acids (e.g., oxalates and malonates) to form Mn^3 +^ chelates, which act as small diffusible oxidants for lignin oxidation (Obinger, 2022). Unlike LiP, which cannot directly attack the dominant non-phenolic structures in lignin polymers and must be stabilized in the presence of a second mediator, MnP aids the oxidation of non-phenolic lignin structures by forming highly reactive radicals (Reddy et al., 2003).

Thus, LiP requires lower conditions and is more efficient at degrading lignin than MnP. Among the two lignin-degrading Bacillus strains, the high-yielding YY-10 LiP enzyme can produce different flavor compounds by degrading more phenolic, non-phenolic compounds compared to the BY-2-yielding MnP. For instance, the concentrations of guaiacol, phenol, and benzyl alcohol in roasted tobacco treated with yy-10 crude enzyme solution were significantly elevated compared to those in the water treatment group. The degradation products of lignin serve as crucial aromatic constituents in roasted tobacco, particularly enhancing its overall aroma profile (Cao et al., 2022). Therefore, in comparison to BY-2, the roasted tobacco treated with YY-10 crude enzyme solution exhibits a more complete degradation of lignin and facilitates its conversion into various flavor compounds. This process ultimately enhances the flavor profile of the roasted tobacco.

Traditional methods of degrading lignin include acid digestion and oxidative degradation, which not only consume more resources but also cause serious environmental pollution. The use of fungi, bacteria, and other microorganisms to degrade lignin has become a hot spot in current research, the growth cycle of fungi is longer than that of bacteria, and its commercial application is still in the research stage. Lignin is cleaved by specific species of bacteria, so bacterial degradation of lignin is more specific than that of fungi. The YY-10 *Bacillus subtilis*, as a typical industrial model microorganism at the food safety level, is widely used in the field of metabolic engineering (Cui et al., 2018). A variety of research strategies and tools are used to construct the *Bacillus subtilis* chassis cell for the efficient synthesis of bioproducts. The metabolic pathways of YY-10 can be further optimized through genetic engineering to increase its production of lignin-degrading enzymes.

The surface YY-10-producing LIPase was found to be highly effective in enhancing the quality of tobacco following treatment across multiple grades. At present, the study of YY-10 is still in the laboratory stage. Subsequent validation of the strain in the industry will be achieved through further pilot fermentation, and the strain will eventually be realized in industrial production, thus solving the problems faced by enterprises. It is anticipated that the proposed methodology will be employed in the treatment of tobacco waste and the exploitation of frangipani.

## 5 Conclusion

Two ligninase-producing *Bacillus strains*, *Bacillus subtilis* YY-10 (GenBank accession no. SUB12195781) and BY-2 (GenBank accession no. SUB12195784), were isolated from the surface of tobacco and produced lignin peroxidase LiP and manganese peroxidase MnP, respectively. Sensory evaluation showed that the fermented crude enzyme solution of *Bacillus subtilis* YY-10 could significantly improve the quality of roasted tobacco. The fermentation crude enzyme solution of *Bacillus subtilis* YY-10 can improve the quality of roasted tobacco, ensuring sufficient aroma, rich aroma, clean smoke, and natural smoke flavor. In contrast, the fermentation solution of the BY-2 strain reduced the aroma level of roasted tobacco and lowered the quality of roasted tobacco.

The lignin and GC–MS assays revealed that the acid-insoluble lignin content of roasted tobacco treated with strain YY-10 fermented crude enzyme solution decreased significantly, and the content of volatile flavor compounds, such as Geranyl acetone, Megastigmatrienone B and Megastigmatrienone C, increased significantly, which could reduce the irritation and enhance the aroma of roasted tobacco, and improve the quality of roasted tobacco. Compared with the BY-2 strain, the expression of genes related to amino acid metabolism, biosynthesis of genetic material, and synthesis of proteins was up-regulated in the YY-10 strain, which promoted the biosynthesis of LiP. Meanwhile, the expression of *copO* and *pxpG*, which are related to the transmembrane transport pathway of metal ions, was up-regulated, which was conducive to the transmutation out of Cu^2+^ as well as transmutation in and out of Fe^2+^, promoting the synthesis of LiP. It suggested that LiP, a lignin-degrading enzyme, played a more significant role in improving the quality of roasted tobacco.

## Data Availability

The datasets presented in this study can be found in online repositories. The names of the repository/repositories and accession number(s) can be found in the article/Supplementary material.
