# Supplementary material for: Comparison of lignin degradation and flavor compound formation in roasted tobacco by two Bacillus subtilis strains
Source: Front Microbiol. 2025 Jan 23;16:1538773. doi: 10.3389/fmicb.2025.1538773 (PMC11801416; doi:10.3389/fmicb.2025.1538773)
Supplement: Supplementary file 1 [file Data_Sheet_1.docx]

Supplementary Material

# Supplementary Data

Supplementary Material should be uploaded separately on submission. Please include any supplementary data, figures and/or tables.

Supplementary material is not typeset so please ensure that all information is clearly presented, the appropriate caption is included in the file and not in the manuscript, and that the style conforms to the rest of the article.

# Supplementary Figures and Tables

For more information on Supplementary Material and for details on the different file types accepted, please see [here](https://www.frontiersin.org/guidelines/author-guidelines#supplementary-material).

TableS1 Table of HC ratio of strains

| strains  serial number | Colony diameter (d1/mm) | Diameter of hydrolysis ring (d2/mm) | HC value | strains  serial number | Colony diameter (d1/mm) | Diameter of hydrolysis ring (d2/mm) | HC value |
| --- | --- | --- | --- | --- | --- | --- | --- |
| WYY-10 | 5.50 | 33.00 | 6.00 | BC-13 | 13.00 | 42.00 | 3.23 |
| WYC-1 | 5.00 | 28.50 | 5.70 | YC-10 | 19.00 | 61.00 | 3.21 |
| WYY-14 | 4.50 | 25.00 | 5.56 | BY-5 | 11.00 | 35.00 | 3.18 |
| YY-10 | 5.00 | 25.00 | 5.00 | WYY-5 | 9.50 | 30.00 | 3.16 |
| YY-5 | 10.00 | 47.00 | 4.70 | YC-6 | 11.50 | 36.00 | 3.13 |
| WYC-2 | 4.00 | 18.00 | 4.50 | YY-14 | 14.00 | 40.50 | 2.89 |
| YY-13 | 10.50 | 45.00 | 4.29 | BC-5 | 17.00 | 47.50 | 2.79 |
| YY-12 | 15.00 | 61.50 | 4.10 | BC-10 | 15.50 | 43.00 | 2.77 |
| YC-9 | 11.50 | 46.50 | 4.04 | BC-18 | 18.00 | 49.00 | 2.72 |
| YC-8 | 10.50 | 41.00 | 3.90 | YC-1 | 12.00 | 30.00 | 2.50 |
| BY-2 | 15.00 | 57.50 | 3.83 | BC-12 | 10.00 | 24.00 | 2.40 |
| YC-7 | 6.00 | 22.00 | 3.67 | BY-10 | 18.00 | 43.00 | 2.39 |
| YY-1 | 14.50 | 53.00 | 3.66 | WYY-1 | 9.00 | 18.00 | 2.00 |
| YC-11 | 8.00 | 28.00 | 3.50 | BC-1 | 18.50 | 22.00 | 1.19 |
| YC-4 | 15.50 | 54.00 | 3.48 | BC-4 | 5.00 | - | - |
| YY-7 | 15.00 | 52.00 | 3.47 | BC-9 | 8.00 | - | - |
| YC-3 | 14.00 | 48.00 | 3.43 | BC-7 | 9.00 | - | - |
| YC-5 | 12.50 | 42.00 | 3.36 | BC-11 | 11.50 | - | - |
| YC-2 | 7.00 | 23.50 | 3.36 | BC-14 | 14.00 | - | - |
| BY-9 | 16.50 | 53.50 | 3.24 | BY-12 | 16.50 | - | - |

Note: HC value = hydrolysis circle diameter/colony diameter, "-" means no hydrolysis circle.

| TableS2 Different flavor compounds in different treatment groups | | | | | |
| --- | --- | --- | --- | --- | --- |
| Serial Number | Name | Flavor Descriptions | Content μg/g | | |
|  |  |  | H_2_O | BY-2 | YY-10 |
| H74 | Neophytadiene | Faint scent | 29.007±1.689^b^ | 37.63±5.362^b^ | 49.398±8.382^a^ |
| H43 | L-Nicotine | None | 102.024±10.871^a^ | 107.781±4.898^a^ | 114.882±18.346^a^ |
| H2 | 2,3-Butanediol | Fruity | 0.878±0.257^b^ | 1.617±0.21^a^ | 0.727±0.143^b^ |
| H56 | 3-(chloromethyl)-2,5,9-trimethylfuro[3,2-g]chromen-7-one | None | 0.184±0.159^b^ | 0.516±0.148^a^ | 0±0^b^ |
| H33 | SPERMINE TETRAHYDROCHLORIDE | None | 1.005±0.049^a^ | 1.688±0.761^a^ | 1.365±0.063^a^ |
| H28 | [Phenethyl alcohol](https://www.chemsrc.com/en/cas/60-12-8_509692.html) | Rose aroma | 0.985±0.072^b^ | 1.484±0.052^b^ | 3.139±1.066^a^ |
| H46 | Damascone | Sweet floral aroma | 0.356±0.032^a^ | 0±0^a^ | 0.475±0.411^a^ |
| H64 | Megastigmatrienone B | Sweet aroma | 1.223±0.868^b^ | 2.226±0.18^b^ | 3.412±0.258^a^ |
| H19 | Benzyl alcohol | Floral aroma | 1.159±0.118^c^ | 2.058±0.486^b^ | 2.825±0.149^a^ |
| H67 | Megastigmatrienone D | Sweet aroma | 1.737±0.135^b^ | 2.01±0.421^b^ | 3.195±0.442^a^ |
| H9 | gamma-Butyrolactone | cream aroma | 0.054±0.054b | 0.261±0.041a | 0±0c |
| H79 | Methyl palmitate | None | 0.724±0.03^c^ | 1.442±0.231^b^ | 2.256±0.293^a^ |
| H45 | Terpinine-4-ol | Fruity | 0.993±0.092^b^ | 1.475±0.364^ab^ | 2.506±0.902^a^ |
| H50 | Geranylacetone | Magnolia aroma | 1.065±0.094^c^ | 1.305±0.058^b^ | 2.109±0.077^a^ |

Note: Different letters indicate significant differences at the 0.05 level (p<0.05)

Supplementary Table S3 QC Data Statistics

The statistical table of quality control data

| Sample  Name | Raw  reads | Raw  Bases | Clean Reads | Clean  Bases | error  Rate (%) Q20 (%) | | Q30 (%) |
| --- | --- | --- | --- | --- | --- | --- | --- |
| YY_10_1 | 73236634 | 11058731734 | 57324008 | 6974810466 | 0.024 | 98.42 | 95.11 |
| YY_10_2 | 26873740 | 4057934740 | 26159802 | 3322222111 | 0.0248 | 98.12 | 94.25 |
| YY_10_3 | 27420140 | 4140441140 | 26729628 | 3341310322 | 0.0247 | 98.17 | 94.42 |
| BY_2_1 | 25472876 | 3846404276 | 24974868 | 3258572961 | 0.0254 | 97.92 | 93.74 |
| BY_2_2 | 60426400 | 9124386400 | 59754716 | 7718238752 | 0.0241 | 98.4 | 95.09 |
| BY_2_3 | 57114284 | 8624256884 | 54988868 | 7180804667 | 0.0234 | 98.73 | 95.76 |
